# Supplementary material for: Phosphoribosylpyrophosphate synthetase as a metabolic valve advances Methylobacterium/Methylorubrum phyllosphere colonization and plant growth
Source: Nat Commun. 2024 Jul 16;15:5969. doi: 10.1038/s41467-024-50342-9 (PMC11252147; doi:10.1038/s41467-024-50342-9)
Supplement: Supplementary file 1 — Supplementary information [file 41467_2024_50342_MOESM1_ESM.pdf]

Supplementary Fig. 1

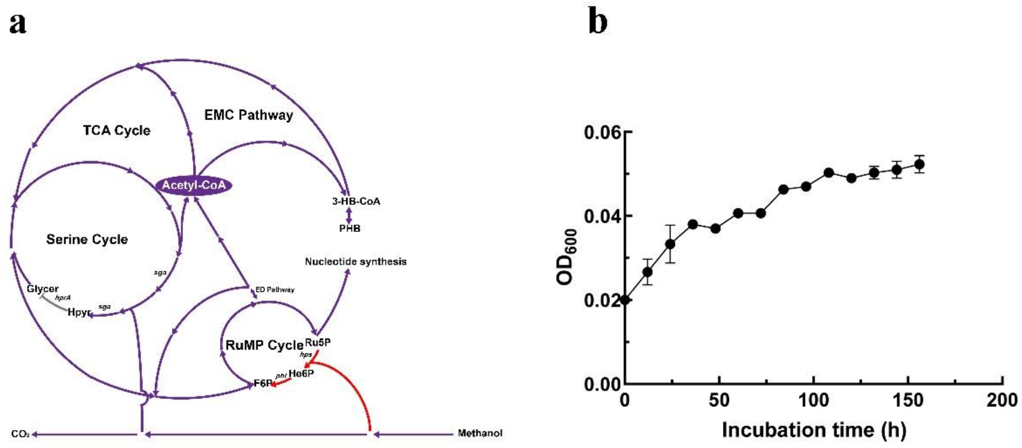

Supplementary Fig. 1 Construction of the rewired strain of RS03. (a) The rewired metabolic cycle for methanol assimilation. Heterologously introduced genes were highlighted in red, and knocked out genes were highlighted in grey. (b) Growth curve of the RS03 strain on methanol at 120 mM. Glycer: glycerate; Hpyr: hydroxypyruvate; Ru5P: ribulose-5-phosphate; He6P: hexulose-6-phosphate; F6P: fructose-6-phosphate; 3-HB-CoA: 3-hydroxybutyryl-CoA; PHB: poly-beta-hydroxybutyrate. Data are presented as mean ± SD (n=6 biologically independent samples with three technical repeats). Source data are provided as a Source Data file.

Supplementary Fig. 2

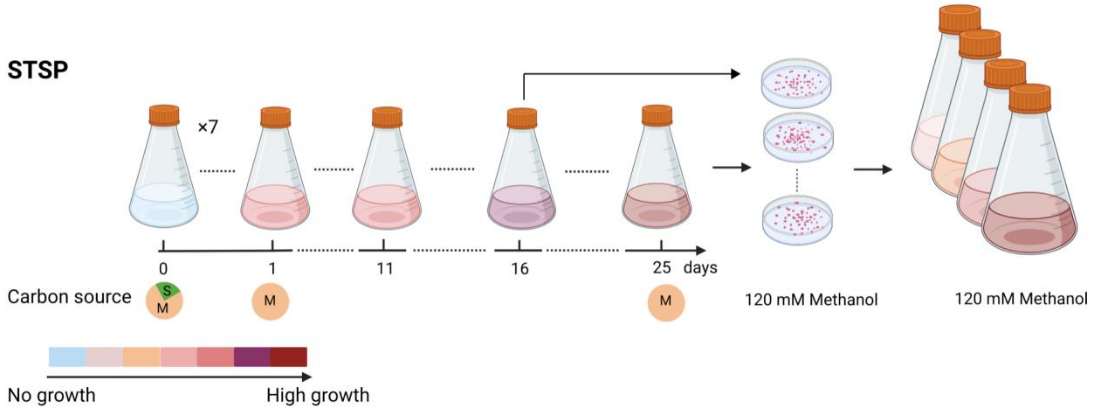

Supplementary Fig. 2 Schematic of the strategy of adaptive laboratory evolution to increase growth fitness of *Mr. extorquens* AM1 at low methanol concentrations. Seven independent lineages of the RS03 strain were subjected to a STSP evolutionary experiment. Cultures were incubated in the medium with 100 mM methanol (M) and 5 mM succinate (S) continuously for 25 days, and the OD_{600} of cultures was determined every two days. On the 16th and 25th day, the cultures were diluted and spread on agar medium with 120 mM methanol. Colonies grown on plates were transferred into liquid medium with 120 mM methanol to obtain the strains stably growing on methanol. The isolated strains were further grown on methanol at low concentrations. The figure was created with Biorender.com.

Supplementary Fig. 3

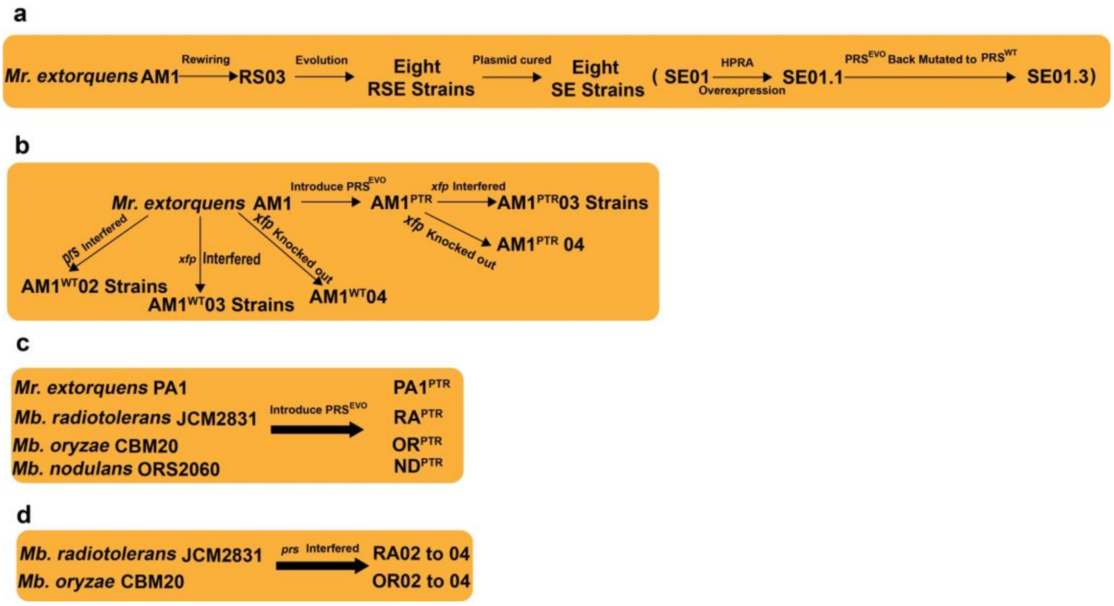

Supplementary Fig. 3 Illustration of the strains obtained in this study. (a) Derivatives of *Mr. extorquens* AM1 evolutionary strains. (b) *prs*/*xfp* interfered or knocked out strains of *Mr. extorquens* AM1 strains. (c) PRS mutated strains of *Mr. extorquens* PA1, *Mb. radiotolerans* JCM2831, *Mb. oryzae* CBM20, and *Mb. nodulans* ORS2060. (d) *prs* interfered strains of *Mb. radiotolerans* JCM2831 and *Mb. oryzae* CBM20.

Supplementary Fig. 4

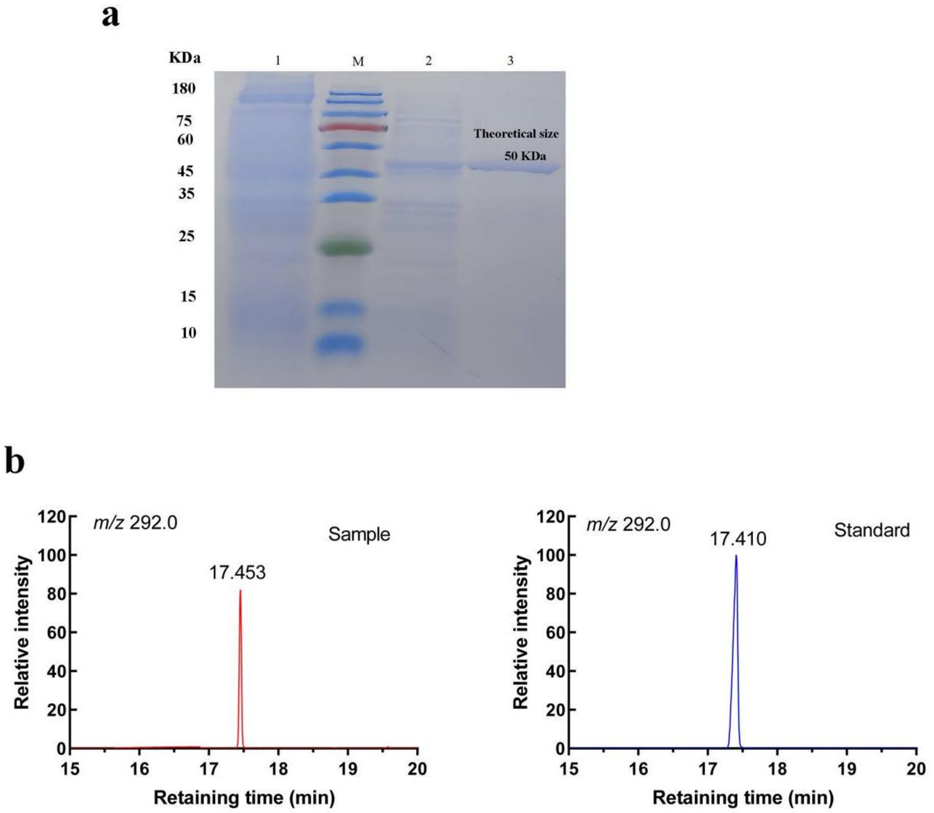

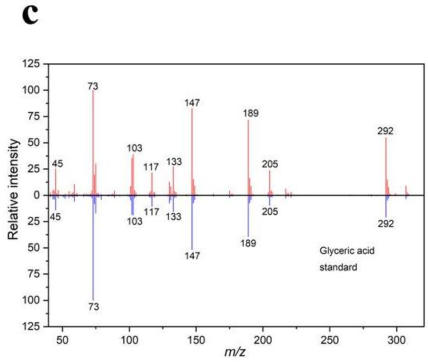

d

*K*m (NADH) (mM)	META_3141	1.38
	HPRA	0.04 Reference1
*K*m (NADPH) (mM)	META_3141	0.42
	HPRA	0.06 Reference1

Supplementary Fig. 4 Enzymatic assay to analyze the function of the hypothetical protein META1_3141. (a) The purified META1_3141 protein was detected by SDS-PAGE. M: Protein marker; 1: Total soluble protein of *E. coli* with empty plasmid pET-32a; 2: Total soluble protein of *E. coli* with recombinant plasmid; 3: Purified recombinant META1_3141. (b) Extracted ion chromatograms of enzymatic sample and glycerate standard. (c) Mass spectrometry of the extracted ion of m/z 292.0 from the enzymatic sample and the glycerate standard. (d) The *K*m values of measured META1_3141 and reported hydroxypyruvate reductase.

Supplementary Fig. 5

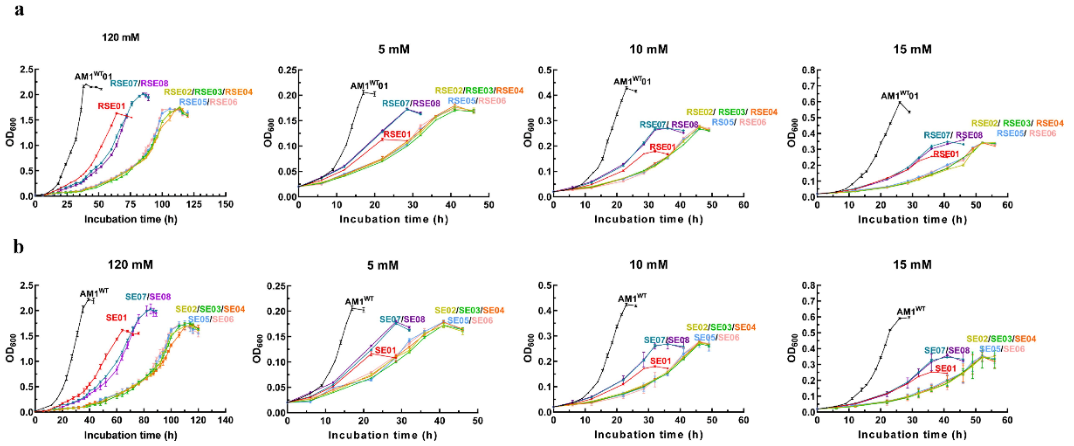

Supplementary Fig. 5 The evolved RSE strains and the SE strains (derivatives of the evolved RSE strains) do not show a growth advantage at low methanol concentrations. (a) Growth curves of eight evolved RSE strains and the AM1^{WT}01 strain (the WT *Mr. extorquens* AM1 with empty plasmid pCM80) on 120 mM methanol, or on low methanol concentrations of 5 mM, 10 mM, and 15 mM. (b) Growth curves of eight SE strains and the AM1^{WT} strain on 120 mM methanol at, or on low methanol concentrations of 5 mM, 10 mM, and 15 mM. Data are presented as mean ± SD (n=6 biologically independent samples with three technical repeats). Source data are provided as a Source Data file.

Supplementary Fig. 6

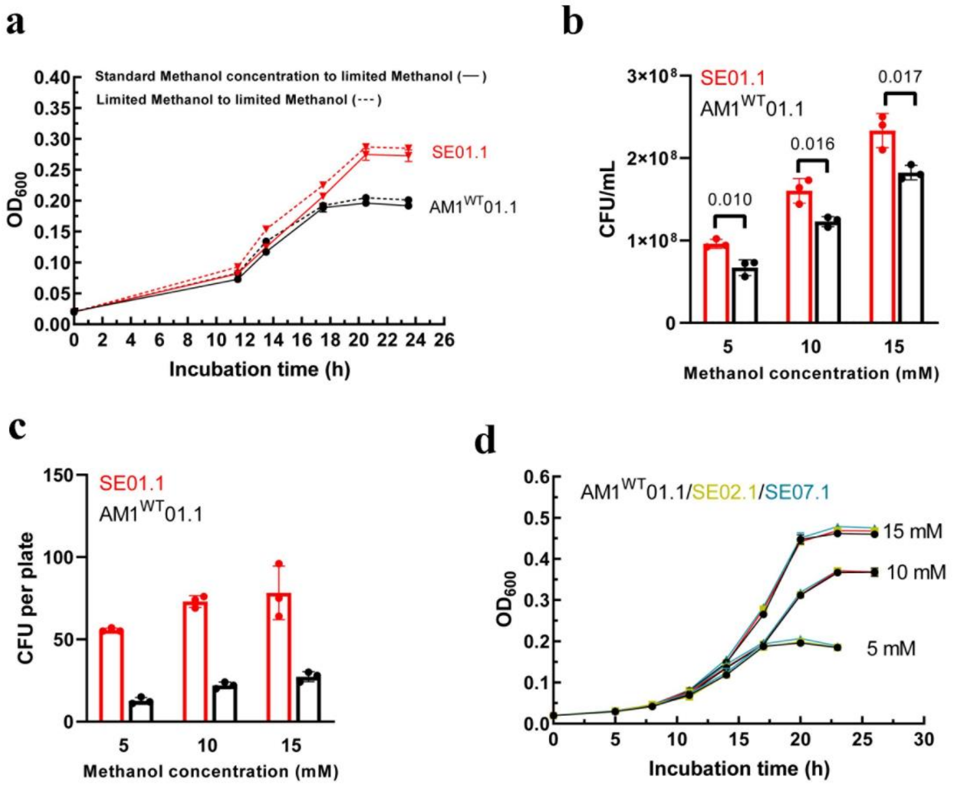

Supplementary Fig. 6 The SE01.1 strain shows a growth advantage at low methanol concentrations, while the SE02.1 and SE07.1 strains do not improve the growth at low methanol concentrations. (a) The growth advantage of the SE01.1 strain transferred from limited-to-limited methanol in liquid media (Solid lines, 5 mM to 5 mM) was the same as the strain transferred from standard-to-limited methanol (Dotted lines, 120 mM to 5 mM). (b) Viable cells of SE01.1 and $AM1^{WT}$01.1 (*Mr. extorquens* AM1-Δ*hprA*-pCM80::*hprA*) at the early stationary growth phase grown at low methanol concentrations. Both strains were grown to the early stationary phase at methanol of 5 mM, 10 mM, and 15 mM, and then spread on the solid agar plate with 120 mM methanol. Colonies were counted after incubating for seven days. (c) Growth advantage of SE01.1 on agar plates with low methanol concentrations. The SE01.1 and $AM1^{WT}$ 01.1 strains were grown to the exponential phase at methanol of 5 mM, 10 mM, and 15 mM, and then were adjusted to the same OD_{600} value and spread on the plate with 5 mM, 10 mM, and 15 mM methanol. Colonies were counted after incubating for three days. (d) The SE02.1 and SE07.1 strains showed the same growth

trend as the AM1^{WT} 01.1 strain. The SE02.1 and SE07.1 strains were obtained by curing the plasmid in the RSE02 and RSE07 strain and then overexpressing *hprA* on pCM80. Data are presented as mean ± SD (For a and d, n=6 biologically independent samples with three technical repeats; for b and c, n=3 biologically independent samples with three technical repeats). Significance was analyzed using a two-tailed t-test analysis. Source data are provided as a Source Data file.

Supplementary Fig. 7

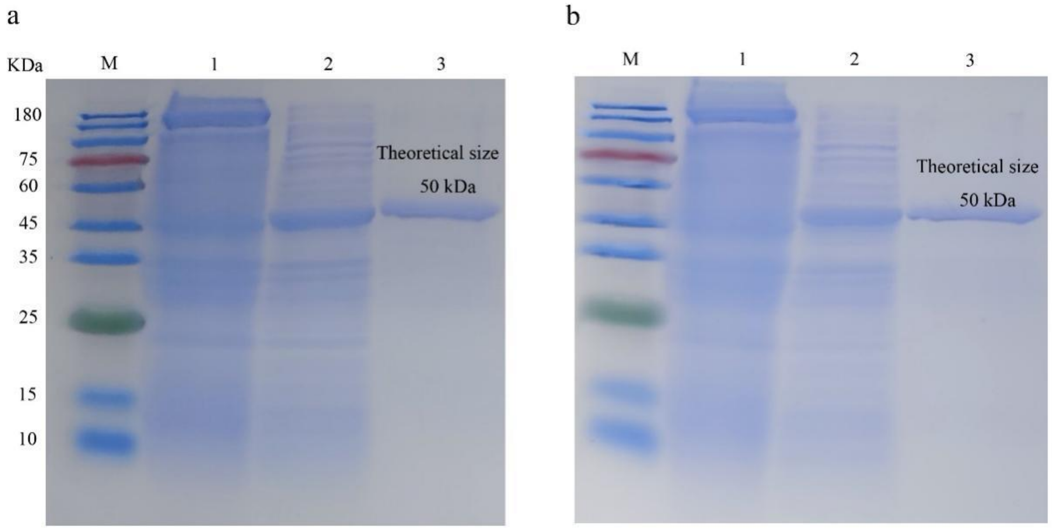

Supplementary Fig. 7 Purification of PRS^{WT} (a) and PRS^{EVO} (b) from *Escherichia coli* BL21 (DE3). M: Protein marker; 1: Total soluble protein of *E. coli* with empty plasmid pET-32a; 2: Total soluble protein of *E. coli* with recombinant plasmid; 3: Purified recombinant PRS.

Supplementary Fig. 8

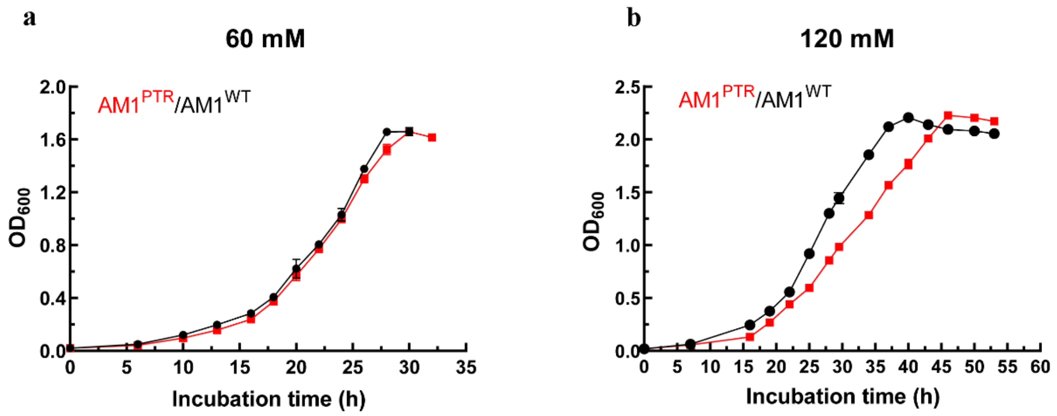

Supplementary Fig. 8 The growth fitness of the AM1^{PTR} strain shows the same trend as the AM1^{WT} strain at 60 mM methanol (a), while its growth appeared slower than the AM1^{WT} strain at 120 mM methanol (b). Data are presented as mean ± SD (n=6 biologically independent samples with three technical repeats). Source data are provided as a Source Data file.

Supplementary Fig. 9

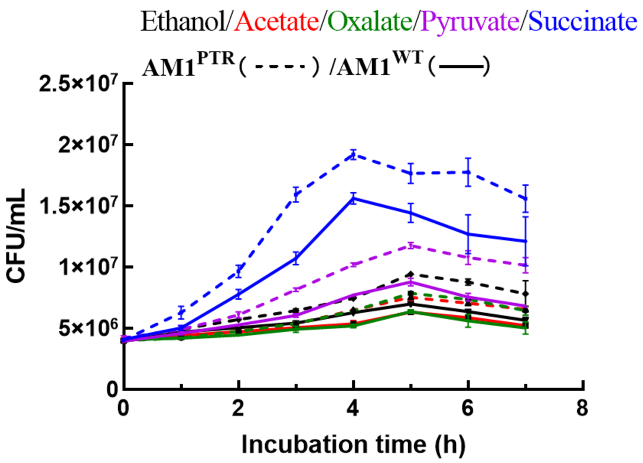

Supplementary Fig. 9 The growth curves of the AM1^{WT} and the AM1^{PTR} strains grown on multi-carbon sources including ethanol, acetate, oxalate, pyruvate, and succinate at 0.1 mM. Data are presented as mean ± SD (n=6 biologically independent samples with three technical repeats). Source data are provided as a Source Data file.

Supplementary Fig. 10

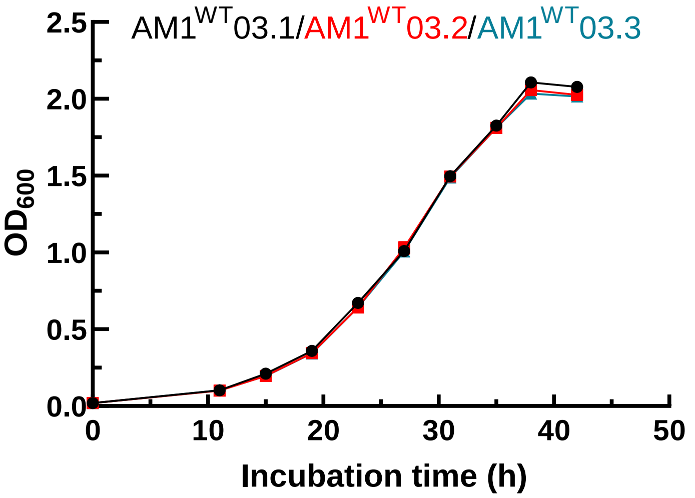

Supplementary Fig. 10 Down-regulation of *xfp2* by CRISPRi does not influence the growth trend of the AM1^{WT} on methanol at 120 mM. AM1^{WT}03.1: the control strain carrying the plasmid pAIO-NT0 (the CRISPRi plasmid without small guide RNA); AM1^{WT}03.2 and AM1^{WT}03.3: the interfered strains carrying the CRISPRi plasmids for targeting different region of *xfp2*. Data are presented as mean ± SD (n=6 biologically independent samples with three technical repeats). Source data are provided as a Source Data file.

Supplementary Fig. 11

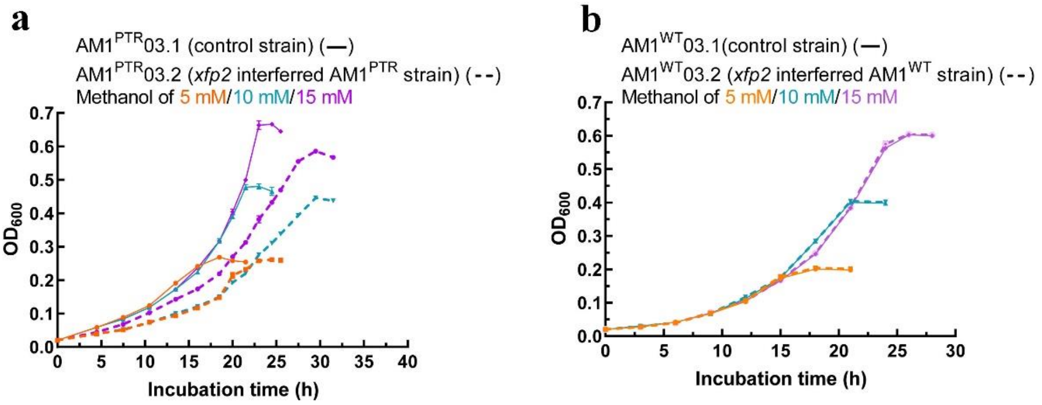

Supplementary Fig. 11 Transcriptional down-regulation of the *xfp*2 gene by CRISPRi decreases the growth advantage of the $AM1^{PTR}$ strain at low methanol concentrations of 5 mM, 10 mM, and 15 mM (a), while it does not affect the growth of the $AM1^{WT}$ strain (b). Data are presented as mean ± SD (n=6 biologically independent samples with three technical repeats). Source data are provided as a Source Data file.

Supplementary Fig. 12

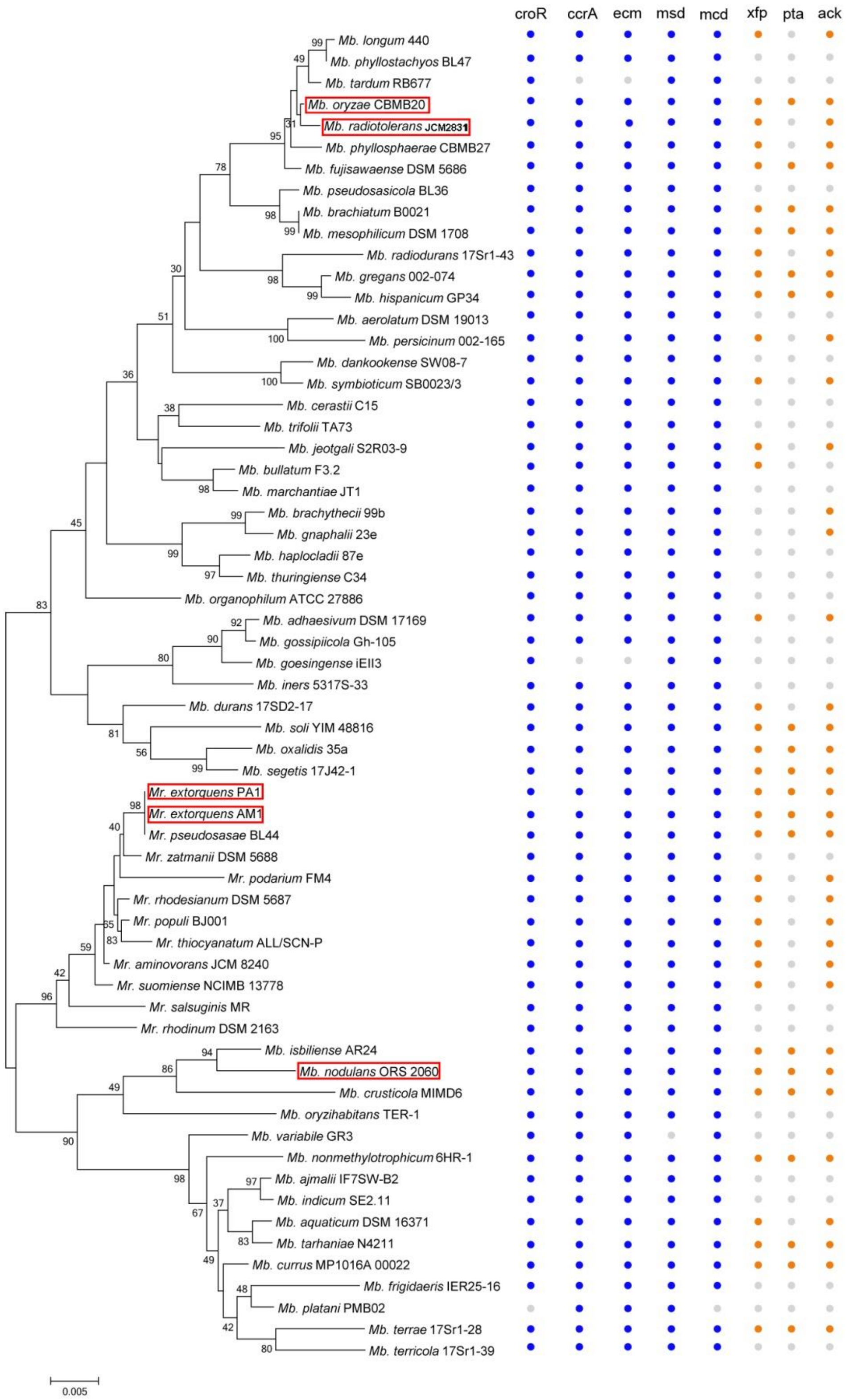

Supplementary Fig. 12 Phylogenetic tree and genome analysis of the members of *Methylobacterium*/*Methylorubrum*. The phylogenetic tree was constructed based on 16S rRNA sequences using the neighbor-joining method with MEGA X. The genomes were analyzed for marker genes of the EMC pathway (*croR*, *ccr*, *ecm*, and *mcd*) and the PKT pathway (*xfp*, *pta*, and *ack*). Strains with genes of *xfp* and *pta* or *ack* were considered to have the PKT pathway. The presence of genes in the EMC pathway were shown in blue dots, and presence of genes in the PKT pathway were shown in orange dots. The absence of genes was shown in grey dots. In this study, strains with a *prs* mutation that possessed growth advantages were marked in red.

Supplementary Fig. 13

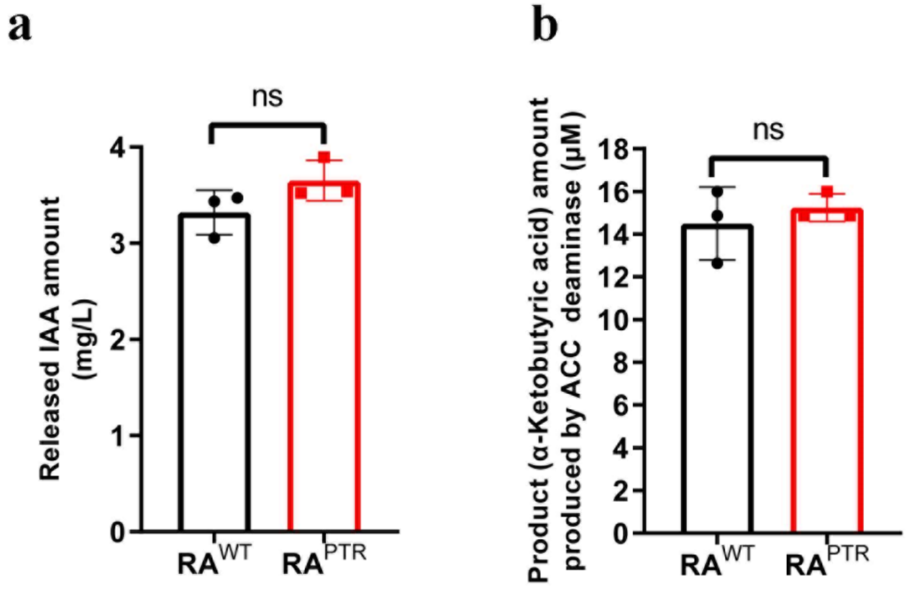

Supplementary Fig. 13 The RA^{PTR} strain produces similar amounts of IAA and ACC deaminase with the RA^{WT} strains. (a) IAA produced by the RA^{WT} and RA^{PTR} strains at OD_{600} of 1.0. (b) The amount of product produced by the RA^{WT} and RA^{PTR} strains at OD_{600} of 1.0 reflected the activity of the ACC deaminase. Data are presented as mean ± SD (n=3 biologically independent samples with three technical repeats). Significance was analyzed using a two-tailed t-test analysis. ns means no significance.

Supplementary Fig. 14

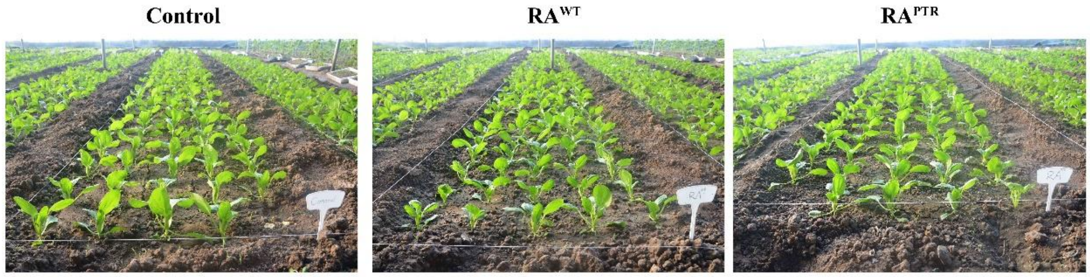

Supplementary Fig. 14 *Brassica chinensis* (commonly known as Pak Choi) is cultivated in agricultural solar greenhouse sprayed with the RA strains.

Supplementary Table 1 Strains studied in this work

Strains	Description	Sources
AM1^{WT}	*Methylorubrum extorquens* AM1 wild-type, pink color, rifamycin-resistant strain,	Reference 74
PA1^{WT}	WT *Mr. extorquens* PA1	Reference 38
RA ^{WT}	WT *Methylobacterium radiotolerans* JCM2831	Reference 39
OR^{WT}	WT *Mb. oryzae* CBM20	Reference 40
ND^{WT}	WT *Mb. nodulans* ORS2060	Reference 39
RS03	*Mr. extorquens* AM1Δ*hpr*A carrying the plasmid pHPL-BM	Reference 29
RSE01 to 08	Evolutionary strains from RS03	This study
SE01 to SE08	RSE01 to RSE 08 cured plasmid pHPL-BM	This study
SE01.1	SE01 carrying plasmid pHPR (pCM80 with *hprA*)	This study
SE02.1	SE02 carrying plasmid pHPR (pCM80 with *hprA*)	This study
SE07.1	SE07 carrying plasmid pHPR (pCM80 with *hprA*)	This study
SE01.3	SE01.1 with *prs*^{EVO} back mutated to *prs*^{WT}	This study
AM1^{WT}01	AM1^{WT} carrying plasmid pCM80	This study
AM1^{WT} 01.1	AM1^{WT} Δ*hpr*A carrying plasmid pHPR	This study
AM1^{PTR}	AM1^{WT} with *prs*^{WT} mutated to *prs*^{EVO}	This study
AM1^{WT} 02.1 to AM1^{WT} 02.4	AM1^{WT} carrying the plasmids pAIO-*prs*-(NT0, NT275, NT398, NT190)	This study
AM1^{WT} 03.1 to AM1^{WT} 03.3	AM1^{WT} carrying the plasmids pAIO-*xfp*-(NT0, NT2034, NT1973)	This study
AM1^{PTR} 03.1, AM1^{PTR} 03.2	AM1^{PTR} carrying the plasmids pAIO-*xfp*-(NT0, NT2034)	This study
OR01 to OR04	*Mb. oryzae* CBM20 carrying the plasmids pAIO-*prs*-(NT0, NT398, NT274, NT190)	This study
RA01 to RA04	*Mb. radiotolerans* JCM2831 carrying the plasmids pAIO -*prs*-(NT0, NT398, NT190, NT274)	This study
PA1^{PTR}	*Mr. extorquens* PA1 with *prs*^{WT} mutated to *prs*^{EVO}	This study
OR^{PTR}	*Mb. oryzae* CBM20 with *prs*^{WT} mutated to *prs*^{EVO}	This study
RA^{PTR}	*Mb. radiotolerans* JCM2831 with *prs*^{WT} mutated to *prs*^{EVO}	This study
ND^{PTR}	*Mb. nodulans* ORS2060 with *prs*^{WT} mutated to *prs*^{EVO}	This study
AM1^{WT} 04	WT *Mr. extorquens* AM1Δ*xfp*	This study
AM1^{PTR} 04	AM1^{PTR} Δ*xfp*	This study
YAIP	WT *Mr. extorquens* AM1Δ*crtI*	Reference 33

Supplementary Table 2 Specific mutations in the genomes of the RSE01, RSE02 and RSE07 strains

Genes	Description	RSE01	RSE02	RSE07
**SNP**				
META1_2146	hypothetical protein	/	/	Q250R (A749G)
META1_2592	DEAD/DEAH box helicase domain protein	P195R (G584C)	/	/
Intergenic region between META1_3141 and META1_3142	META1_3141: Ketopantoate reductase ApbA/PanE<br>META1_3142: hypothetical protein		C3280661A	
META1_3302	LL-diaminopimelate aminotransferase	/	/	R295C (C883T)
META1_4249	ribose-phosphate pyrophosphokinase	D38N (G112A)	/	/
**Insertion/Deletion**				
META1_0458	hypothetical protein		I1bp C482893	
META1_0557	methyl-accepting chemotaxis receptor/sensory transducer		I1bp C580985	
META1_0574	conserved hypothetical protein with putative ATP-dependent helicase domain		I1bp T601196	
META1_0740	DUF3572 domain-containing protein		Δ1bp G770863	
META1_2262	patatin-like phospholipase		Δ1bp C350	
META1_2592	DEAD/DEAH box helicase domain protein	I2bp, Δ3bp	/	/
META1_2909	maltose alpha-D-glucosyltransferase		Δ1bp	
META1_3022	DEAD/DEAH box helicase		Δ1bp	
META1_3892	glutathione transferase		Δ5bp	
META1_3908	leucyl aminopeptidase	/	I12bp	/
META1_3990	hypothetical protein	/	/	I2bp, I2bp, Δ2bp
META1_3992	hypothetical protein	/	/	I1bp, Δ3bp
META1_4005	hypothetical protein	/	Δ1bp Δ3bp	/
Intergenic region between META1_4007 and META1_4009	META1_4007: hypothetical protein<br>META1_4009: hypothetical protein	/	Δ1bp	/
META1_4009	hypothetical protein	/	I2bp Δ2bp	/
Intergenic region between META1_4710 and META1_4711	META1_4710: hypothetical protein<br>META1_4711: hypothetical protein	/	Δ1bp	/
PlasmidpCM80-PmxaF	Promoter and RBS	△366bp	/	/

Supplementary Table 5 Plasmids used in this study

Plasmids	Description	Sources
pCM80	vector used for gene expression in *Mr. extorquens*; promoter, PmxaF; antibiotics, TetR, KmR	Reference 65
pCM433	*sacB*-based allelic exchange vector; antibiotics, ApR, CmR, TetR	Reference 64
pHPL-BM	pCM80 derivative contained *hpsBM* and *phiBM* connected with linker under the control of constitutive promoter PmxaF	Reference 29
pHPR	pCM80 derivative contained *Mr. extorquens* AM1 *hprA*	This study
pPRSW	*Mr. extorquens* AM1 *prs*^{WT} in pCM433	This study
pPRSE	*Mr. extorquens* AM1 *prs*^{EVO} in pCM433	This study
pET-PRSW	pET-32a derivative contained *Mr. extorquens* AM1 *prs*^{WT}	This study
pET-PRSE	pET-32a derivative contained *Mr. extorquens* AM1 *prs*^{EVO}	This study
pAIO	pCM80 derivative with dcas9 and sgRNA expression cassette; dcas9 and sgRNA under the control of inducible promoter PR/tetO and PmxaF-g, respectively	Reference 33
pKNXFP	*Mr. extorquens* AM1 *xfp* deletion in pCM433	This study
pPRSE-PA1	*Mr. extorquens* PA1 *prs* ^{EVO} in pCM433	This study
pPRSE-OR	*Mb. oryzae* CBM20 *prs*^{EVO} in pCM433	This study
pPRSE-RA	*Mb. radiotolerans* JCM2831 *prs* ^{EVO} in pCM433	This study
pPRSE-ND	*Mb. nodulans* ORS2060 *prs* ^{EVO} in pCM433	This study

Supplementary Table 7 Soil composition in the solar greenhouse

Items	Contents
Moisture content	13.8%
pH	7.40
Hydrolyzable nitrogen	369 mg/kg
Organic matter	29.6 g/kg
Chloride ion	0.484 g/kg
Available phosphorus	56.3 mg/kg
EC Value	128 mS/m
Soil infiltration rate	0.68 mm/min
Available potassium	663 mg/kg
Available calcium	650 mg/kg
Available copper	2.26 mg/kg
Available ion	4.74 mg/kg
Available magnesium	988 mg/kg
Available manganese	1.52 mg/kg
Available zinc	5.86 mg/kg
Cation exchange capacity	8.2 $cmol^{+}$/kg
Available sulfur	10.6 mg/kg
Available molybdenum	0.12 mg/kg

Reference

1. Chistoserdova, L.V. & Lidstrom, M.E. Purification and characterization of hydroxypyruvate reductase from the facultative methylotroph *Methylobacterium extorquens* AM1. *J Bacteriol*, **173**, 7228-7232 (1991).
