# Supplementary material for: Phosphoribosylpyrophosphate synthetase as a metabolic valve advances Methylobacterium/Methylorubrum phyllosphere colonization and plant growth
Source: Nat Commun. 2024 Jul 16;15:5969. doi: 10.1038/s41467-024-50342-9 (PMC11252147; doi:10.1038/s41467-024-50342-9)
Supplement: Supplementary file 3 — Description of Additional Supplementary Files [file 41467_2024_50342_MOESM3_ESM.pdf]

Description of Additional Supplementary Files

Supplementary Data 1: The comparison of metabolite pools between Mr. extorquens strains of AM1PTR and AM1WT grown on 10 mM methanol. Each sample had three biological replicates. Significance was analyzed using a two-tailed t-test analysis.

Supplementary Data 2: The comparison of transcriptional levels of genes involved in the central methlyotrophic pathways between Mr. extorquens strains of AM1PTR and AM1WT grown on 10 mM methanol. Each sample had three biological replicates with five technical repeats. Significance was analyzed using a two-tailed t-test analysis.

Supplementary Data 3: Primers used in this study.
