# Supplementary material for: Phosphoribosylpyrophosphate synthetase as a metabolic valve advances Methylobacterium/Methylorubrum phyllosphere colonization and plant growth
Source: Nat Commun. 2024 Jul 16;15:5969. doi: 10.1038/s41467-024-50342-9 (PMC11252147; doi:10.1038/s41467-024-50342-9)
Supplement: Supplementary file 7 — Reporting Summary [file 41467_2024_50342_MOESM7_ESM.pdf]

nature portfolio

Corresponding author(s): Song Yang

Last updated by author(s): 2024-6-15

Reporting Summary

Nature Portfolio wishes to improve the reproducibility of the work that we publish. This form provides structure for consistency and transparency in reporting. For further information on Nature Portfolio policies, see our Editorial Policies and the Editorial Policy Checklist.

Statistics

For all statistical analyses, confirm that the following items are present in the figure legend, table legend, main text, or Methods section.

n/a	Confirmed	
☐	☒	The exact sample size (*n*) for each experimental group/condition, given as a discrete number and unit of measurement
☐	☒	A statement on whether measurements were taken from distinct samples or whether the same sample was measured repeatedly
☐	☒	The statistical test(s) used AND whether they are one- or two-sided<br>*Only common tests should be described solely by name; describe more complex techniques in the Methods section.*
☒	☐	A description of all covariates tested
☐	☒	A description of any assumptions or corrections, such as tests of normality and adjustment for multiple comparisons
☐	☒	A full description of the statistical parameters including central tendency (e.g. means) or other basic estimates (e.g. regression coefficient) AND variation (e.g. standard deviation) or associated estimates of uncertainty (e.g. confidence intervals)
☐	☒	For null hypothesis testing, the test statistic (e.g. F, t, r) with confidence intervals, effect sizes, degrees of freedom and P value noted<br>*Give P values as exact values whenever suitable.*
☒	☐	For Bayesian analysis, information on the choice of priors and Markov chain Monte Carlo settings
☒	☐	For hierarchical and complex designs, identification of the appropriate level for tests and full reporting of outcomes
☒	☐	Estimates of effect sizes (e.g. Cohen's *d*, Pearson's *r*), indicating how they were calculated

Our web collection on statistics for biologists contains articles on many of the points above.

Software and code

Policy information about availability of computer code

Data collection	Specific growth rates were collected by Curve Fitter (4.5.64); The data of genome sequencing were collected by PacBio Sequel plateform, and Illumina Novaseq PE150; The three-dimensional structure of protein were collected by α-fold2 (http://alphafold. ebi.ac.uk/); Data of LC-MS were collected using an UHPLC (1290 Infinity LC; Agilent Technologies) coupled to a QTRAP MS (6500+, AB SCIEX); Data of GS-MS were collected using GC17A with QP-2020 mass spectrometer.
Data analysis	Data of genome sequnecing analysis: SAMTOOLS (v.0.1.18), CNVnator (v.0.3); Phylogenetic analysis: MEGA X; Statistical analysis: Microsoft Excel 2021; Data of RT-qPCR analysis: Quantstudio TM Design and Analysis Software (v1.4.3);Data of GC-MS analysis: GCMS LabSolutions (v4.45SP1); Data of LC-MS analysis: MultiQuant (v3.0.3).

For manuscripts utilizing custom algorithms or software that are central to the research but not yet described in published literature, software must be made available to editors and reviewers. We strongly encourage code deposition in a community repository (e.g. GitHub). See the Nature Portfolio guidelines for submitting code & software for further information.

Data

Policy information about availability of data

All manuscripts must include a data availability statement. This statement should provide the following information, where applicable:

- Accession codes, unique identifiers, or web links for publicly available datasets
- A description of any restrictions on data availability
- For clinical datasets or third party data, please ensure that the statement adheres to our policy

Whole-genome sequencing data of the strains RSE01, RSE02, and RSE07 have been deposited at the National Center for Biotechnology Information under Bioproject number PRJNA1031812 (http://www.ncbi.nlm.nih.gov/bioproject/?term=PRJNA103812). Source data are provided with this paper.

Research involving human participants, their data, or biological material

Policy information about studies with human participants or human data. See also policy information about sex, gender (identity/presentation), and sexual orientation and race, ethnicity and racism.

Reporting on sex and gender	Not applicable
Reporting on race, ethnicity, or other socially relevant groupings	Not applicable
Population characteristics	Not applicable
Recruitment	Not applicable
Ethics oversight	Not applicable

Note that full information on the approval of the study protocol must also be provided in the manuscript.

Field-specific reporting

Please select the one below that is the best fit for your research. If you are not sure, read the appropriate sections before making your selection.

☒ Life sciences ☐ Behavioural & social sciences ☐ Ecological, evolutionary & environmental sciences

For a reference copy of the document with all sections, see nature.com/documents/nr-reporting-summary-flat.pdf

Life sciences study design

All studies must disclose on these points even when the disclosure is negative.

Sample size	To assess the ability of Methylobacterium/Methylorubrum to colonize on Arabidopsis thaliana leaves under a gnotobiotic system, a minimum of 25 plant individuals were evaluated; To assess the ability of Methylobacterium/Methyloruburm to colonize on A.thaliana leaves in growth chambers, more than 40 plant individuals were evaluated; To assess the ability of Methylobacterium/Methylorubrum to promote plant growth in a solar greenhouse, one thousand plant individuals were evaluated.
Data exclusions	No data were excluded in this work.
Replication	At least three independent experiments were performed to verify our results. All of the attempts were successful.
Randomization	The strains were randomly allocated into independent experiments.
Blinding	Researchers were blinded to group allocation during data collection and analysis process.

Reporting for specific materials, systems and methods

We require information from authors about some types of materials, experimental systems and methods used in many studies. Here, indicate whether each material, system or method listed is relevant to your study. If you are not sure if a list item applies to your research, read the appropriate section before selecting a response.

Materials & experimental systems

n/a	Involved in the study	
☒	☐	Antibodies
☒	☐	Eukaryotic cell lines
☒	☐	Palaeontology and archaeology
☒	☐	Animals and other organisms
☒	☐	Clinical data
☒	☐	Dual use research of concern
☐	☒	Plants

Methods

n/a	Involved in the study	
☒	☐	ChIP-seq
☒	☐	Flow cytometry
☒	☐	MRI-based neuroimaging

Dual use research of concern

Policy information about dual use research of concern

Hazards

Could the accidental, deliberate or reckless misuse of agents or technologies generated in the work, or the application of information presented in the manuscript, pose a threat to:

No	Yes	
☒	☐	Public health
☒	☐	National security
☒	☐	Crops and/or livestock
☒	☐	Ecosystems
☒	☐	Any other significant area

Experiments of concern

Does the work involve any of these experiments of concern:

No	Yes	
☒	☐	Demonstrate how to render a vaccine ineffective
☒	☐	Confer resistance to therapeutically useful antibiotics or antiviral agents
☒	☐	Enhance the virulence of a pathogen or render a nonpathogen virulent
☒	☐	Increase transmissibility of a pathogen
☒	☐	Alter the host range of a pathogen
☒	☐	Enable evasion of diagnostic/detection modalities
☒	☐	Enable the weaponization of a biological agent or toxin
☒	☐	Any other potentially harmful combination of experiments and agents

Plants

Seed stocks	Seeds of Arabidopsis thaliana Col0 were stored in the laboratory; Seeds of Brassica chinensis, Cucumis melo and Cucumis stivus were purchased from Shandong Shouhe Seed Industry Co., Ltd, China.
Novel plant genotypes	Novel plant genotypes were not applicable for the study, since no novel plant genotypes were performed.
Authentication	Authentication was not applicable for the study.
